# Phase III trial of short-course radiotherapy followed by CAPOXIRI versus CAPOX in locally advanced rectal cancer: the ENSEMBLE trial

**DOI:** 10.1016/j.esmogo.2023.08.002

**Published:** 2023-11-07

**Authors:** J. Watanabe, Y. Kagawa, K. Chida, K. Ando, D. Kotani, K. Oba, H. Bando, H. Hojo, S. Shimamoto, S. Sakashita, T. Kuwata, T. Tsuboyama, N. Hosomi, M. Uemura, K. Uehara, M. Ito, E. Oki, I. Takemasa, E. Misugi, G. Sledge, K. Sumani, S. Imoto, T. Kato, T. Yoshino

**Affiliations:** 1 Department of Surgery, Gastroenterological Center, Yokohama City University Medical Center, Yokohama; 2 Department of Gastroenterological Surgery, Osaka General Medical Center, Osaka; 3 Department of Colorectal Surgery, National Cancer Center Hospital East, Chiba; 4 Department of Gastroenterology and Gastrointestinal Oncology, National Cancer Center Hospital East, Chiba; 5 Department of Biostatistics, School of Public Health, The University of Tokyo, Tokyo; 6 Translational Research Support Section, National Cancer Center Hospital East, Chiba; 7 Department of Radiation Oncology, National Cancer Center Hospital East, Chiba; 8 Department of Radiology, Osaka General Medical Center, Osaka; 9 Division of Pathology, National Cancer Center Hospital East, Chiba; 10 Department of Pathology and Clinical Laboratories, National Cancer Center Hospital East, Chiba; 11 Department of Diagnostic and Interventional Radiology, Osaka University Graduate School of Medicine, Osaka; 12 Department of Diagnostic Radiology, Osaka General Medical Center, Osaka; 13 Department of Gastroenterological Surgery, Osaka University Graduate School of Medicine, Osaka; 14 Department of Gastroenterological Surgery, Nippon Medical School Hospital, Tokyo; 15 Department of Surgery and Science, Graduate School of Medical Sciences, Kyushu University, Fukuoka; 16 Department of Surgery, Surgical Oncology and Science, Sapporo Medical University, Sapporo; 17 YCU Center for Novel and Exploratory Clinical Trials, Yokohama City University Hospital, Yokohama, Japan; 18 Caris MPI, Inc. d/b/a/ Caris Life Sciences, Irving, USA; 19 Department of Laboratory Medicine, National Cancer Center Hospital, Tokyo; 20 Division of Health Medical Intelligence, Human Genome Center, The Institute of Medical Science, The University of Tokyo, Tokyo; 21 Department of Surgery, National Hospital Organization Osaka National Hospital, Osaka, Japan

**Keywords:** total neoadjuvant therapy, locally advanced rectal cancer, non-operative management, triplet, randomized controlled trial, whole-genome sequencing

## Abstract

The two key concerns in treating locally advanced rectal cancer (LARC) are as follows: (i) prolonging survival by reducing distant metastases and (ii) maintaining anorectal function and quality of life in surviving patients by safely avoiding rectal resection. To resolve these issues, in recent years, total neoadjuvant therapy (TNT), a preoperative combination of chemoradiotherapy or short-course radiotherapy (SCRT) and systemic chemotherapy, has been developed as a multidisciplinary treatment of LARC. There have been no prospective studies on consolidation triplet versus doublet regimens after SCRT. This randomized phase III trial (the ENSEMBLE trial) aims to test the superiority of consolidation irinotecan, capecitabine, and oxaliplatin over capecitabine and oxaliplatin after SCRT as TNT for LARC. The primary endpoint will be organ preservation-adapted disease-free survival in the intention-to-treat population. Moreover, no predictive biomarkers have been established for LARC. Therefore, to explore the predictive biomarkers for estimating the response to TNT and non-operative management, we planned translational research using multi-omics data, including genomic profiling with whole-genome/transcriptome sequencing of tissue and blood samples, liquid biopsy, radiomics, digital pathology, clinical features by deep learning with artificial intelligence.

## Highlights

• The two key concerns in treating LARC are prolonging survival and maintaining QoL by safely avoiding rectal resection.
• There have been no prospective studies of consolidation triplet versus doublet regimens following short course radiotherapy.
• This RCT aimed to test the superiority of consolidation triplet (CAPOXIRI) vs. doublet (CAPOX) regimen after SCRT.
• The primary endpoint is organ preservation-adapted disease-free survival in the intention-to-treat population.
• We planned translational research using multi-omics data and the establishment of NOM predictors by deep learning.

## Graphical abstract

**Figure:**
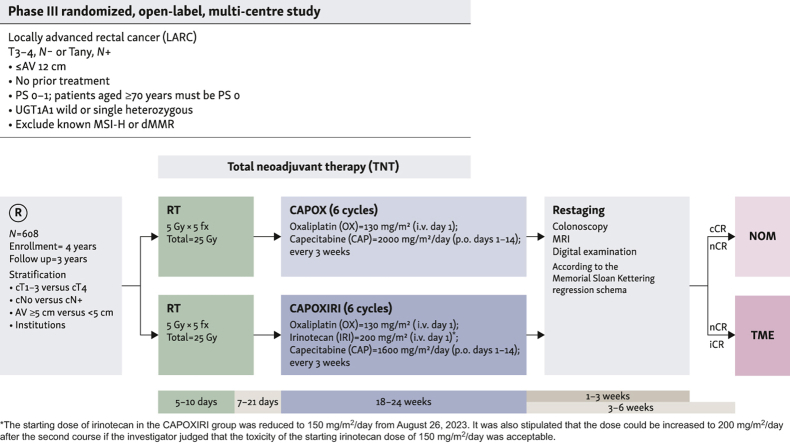

**Table:** Specifications table

Subject area	Medicine and Dentistry
**More specific subject area**	Preoperative treatment of locally advanced rectal cancer.
**Name of your trial in progress**	The ENSEMBLE trial
**Reagents/tools**	The standard-care group receives short-course radiation therapy (5 × 5 Gy) followed by six cycles of CAPOX (capecitabine 1000 mg/m^2^ orally twice daily on days 1-14, oxaliplatin 130 mg/m^2^ intravenously (i.v.) on day 1, every 3 weeks). The experimental-care group receives short-course radiation therapy (5 × 5 Gy) followed by six cycles of CAPOXIRI (capecitabine 800 mg/m^2^ orally twice daily on days 1-14, oxaliplatin 130 mg/m^2^ i.v. on day 1, and irinotecan 200 mg/m^2^ i.v. on day 1∗, every 3 weeks). ∗The starting dose of irinotecan in the CAPOXIRI group was reduced to 150 mg/m^2^/day from August 26, 2023. It was also stipulated that the dose could be increased to 200 mg/m^2^/day after the second course if the investigator judged that the toxicity of the starting irinotecan dose of 150 mg/m^2^/day was acceptable.
**Trial design**	The ENSEMBLE trial is a multicenter, open-label, randomized phase III trial. Eligible patients will be randomly assigned in a 1 : 1 ratio to receive SCRT (5 × 5 Gy) followed by six cycles of CAPOXIRI (experimental-care arm) or CAPOX (standard-care arm). Patients are eligible if they are aged ≥18 years with an Eastern Cooperative Oncology Group performance status of 0 or 1 and had a histologically confirmed diagnosis of adenocarcinoma of the rectum (≤12 cm from the anal verge), cStage II (cT3-4N0M0), or cStage III (cTanyN1-2M0). The primary endpoint will be organ preservation-adapted disease-free survival in the intention-to-treat population. To detect a decrease in 3-year cumulative probability of organ preservation-adapted DFS from 75.0% to 81.7%, corresponding to a target hazard ratio of 0.70, 608 patients (196 events) would achieve 70% power at a two-sided α significance level of 0.05, with a 4-year enrollment period and 3-year follow-up period.
**Trial registration**	jRCTs031220342 and NCT05646511
**Ethics**	The current study has been approved by the National Cancer Center Hospital East Certified Review Board (ID: CRB3180009), and permission to conduct the study has been obtained from the management of all participating facilities. Written informed consent for participation will be obtained from all participants.
**Value of the Trial in Progress**	• This is the first phase III trial to evaluate the effects of preoperative chemotherapy intensity following short-course radiotherapy on long-term outcomes and the proposed treatment will improve the survival and preserve the quality of life of patients with locally advanced rectal cancer. • We will establish the predictive biomarkers for estimating the response to total neoadjuvant therapy and non-operative management from translational research using multi-omics data, including genomic profiling with whole-genome/transcriptome sequencing of tissue and blood samples, liquid biopsy, radiomics, digital pathology, clinical features by deep learning with artificial intelligence.

## Background and rationale

The two key concerns in treating locally advanced rectal cancer (LARC) are as follows: (i) prolonging survival by reducing distant metastases and (ii) maintaining anorectal function and quality of life (QoL) in surviving patients by safely avoiding rectal resection. To resolve these issues, in recent years, total neoadjuvant therapy (TNT), a preoperative combination of chemoradiotherapy (CRT) or short-course radiotherapy (SCRT) and systemic chemotherapy, has been developed as a multidisciplinary treatment of LARC. There have been no prospective studies on consolidation triplet versus doublet regimens after SCRT. This randomized phase III trial (the ENSEMBLE trial) aims to test the superiority of consolidation irinotecan, capecitabine, and oxaliplatin over capecitabine and oxaliplatin after SCRT as TNT for LARC (More on background and rationale is available at Supplementary Material, available at https://doi.org/10.1016/j.esmogo.2023.08.002).

## Objectives

This trial aims to demonstrate that the intensity of consolidation chemotherapy (CNCT) after SCRT contributes to the improvement of long-term outcomes. In addition, we will develop a trans-omics deep learning (DL) model using genomic data from whole-genome sequencing (WGS), transcriptome sequencing (TS), liquid biopsy, colonoscopy, pelvic magnetic resonance imaging (MRI) at each point, and clinicopathological features to identify patients who will benefit from non-operative management (NOM) after TNT.

## Methods

### Trial design and treatment

**Figure 1:**
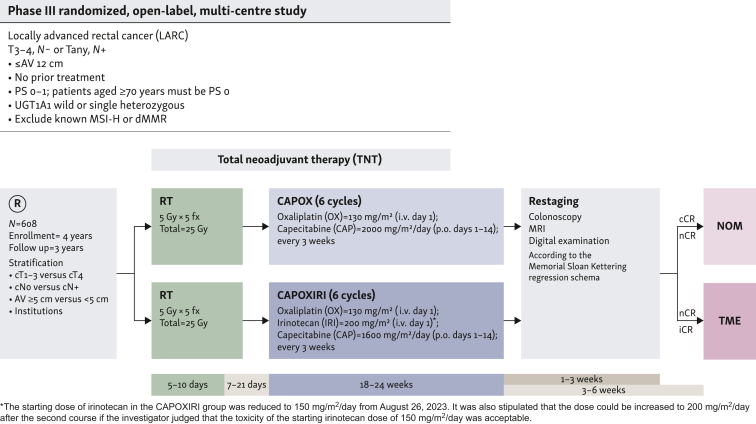
**Trial design.** AV, anal verge; cCR, clinical complete response; i.v., intravenous; dMMR, defective mismatch repair; iCR, incomplete CR; i.v., intravenous; LARC, locally advanced rectal cancer; MRI, magnetic resonance imaging; MSI-H, microsatellite instability-high; nCR, near CR; NOM, non-operative management; PS, Eastern Cooperative Oncology Group performance status; TME, total mesorectal excision; TNT, total neoadjuvant therapy.

The ENSEMBLE trial is a multicenter, open-label, randomized phase III trial (Figure 1). Case registration will be carried out using an electronic data capture system (Viedoc®), and eligible patients will be randomly assigned in a 1 : 1 ratio to receive SCRT (5 × 5 Gy) followed by six cycles of CAPOXIRI (capecitabine 800 mg/m^2^ orally twice daily on days 1-14, oxaliplatin 130 mg/m^2^ intravenously (i.v.) on day 1, and irinotecan 200 mg/m^2^ i.v. on day 1, every 3 weeks) (experimental-care arm) or CAPOX (capecitabine 1000 mg/m^2^ orally twice daily on days 1-14, oxaliplatin 130 mg/m^2^ i.v. on day 1, every 3 weeks) (standard-care arm). Randomization will be carried out by the minimization method using institution strata, clinical T stage (cT1-3 versus cT4), clinical N status (cN– versus cN+), and distance from the anal verge (<5 cm versus ≥5 cm). Preoperative chemotherapy should be initiated 1-3 weeks (allowable up to 4 weeks) after the last day of SCRT, and those in both arms must undergo tumor assessment and restaging based on colonoscopy, pelvic MRI, and digital findings according to the Memorial Sloan Kettering Regression Schema^1^ within 1-3 weeks after the completion of preoperative chemotherapy (last day of capecitabine administration) or the date of discontinuation. Patients with incomplete complete response (iCR) will undergo total mesorectal excision (TME), whereas those with clinical complete response (cCR) will be treated with NOM or near complete response (nCR) and can undergo TME or NOM at the investigator’s discretion under the recommendation of an assessment by the designated NOM central committee composed of experts (radiologists, oncologists, and gastrointestinal surgeons) using a web viewer (Restlogy Inc.). NOM and TME must be carried out within 3-6 weeks of the completion of preoperative chemotherapy (the last day of capecitabine administration) or the date of discontinuation, respectively.

### Eligibility criteria

The eligibility criteria are presented in Table 1. Briefly, patients are eligible if they are aged ≥18 years with an Eastern Cooperative Oncology Group performance status (ECOG-PS) of 0 or 1 and had a histologically confirmed diagnosis of adenocarcinoma of the rectum (≤12 cm from the anal verge), cStage II (cT3-4N0M0), or cStage III (cTanyN1-2M0) based on Union for International Cancer Control (UICC) TNM classification (8th edition).^2^

**Table 1:** Inclusion and exclusion criteria

Inclusion criteria
• Patients who must read, agree to, and sign a statement of informed consent before participation in this study
• Histologically confirmed diagnosis of adenocarcinoma of the rectum
• Distance from the anal verge <12 cm
• Clinical stage II (T3-4, N–)∗ or stage III (any T, N+) according to the tumor–node–metastasis 8th edition ∗Only patients with 5 cm < AV < 10 cm, T3a/bN0M0, EMVI–, MRF clear, and 10 cm < AV < 12 cm, T3a/bN0-1M0, EMVI–, MRF clear who refuse surgery will be eligible
• No evidence of distant metastases
• Rectal tumor at baseline that would be considered to require complete total mesorectal excision
• No prior pelvic radiation therapy
• No prior chemotherapy or surgery for rectal cancer
• Aged ≥18 years
• ECOG performance status 0-1 (if patient aged ≥70 years, ECOG PS 0)
• UGT1A1 wild or single hetero
• Adequate hematological function, defined as absolute neutrophil count >1500/μl, platelet count >100 000/μl, and hemoglobin >9.0 g/dl
• Adequate hepatic function, defined as total bilirubin <2.0 mg/dl and aspartate transaminase/alanine transaminase levels <100 IU/l
• Adequate renal function, defined as calculated creatinine clearance >30 ml/min using the Cockcroft and Gault formula

Exclusion criteria
• Major surgery within 28 days before the initiation of protocol treatment, excluding ileostomy/colostomy and central venous port placement
• Complications or history of severe lung disease (e.g. interstitial pneumonia, pulmonary fibrosis, severe emphysema)
• Patients who underwent placement of a self-expandable metal stent
• Patients who are unable to undergo magnetic resonance imaging
• Two or more synchronous colorectal cancers (multiple cancer)∗ ∗Patients with clinical stage Tis or T1a colorectal cancer judged to be treated by local treatment may be included in this study only when it is confirmed that the cancer has been completely resected by local treatment
• Active double cancer∗ ∗Patients, however, with a relapse-free survival period of >5 years or patients with skin basal cell carcinoma, spinocellular carcinoma, superficial bladder cancer, or cervical cancer that has been treated by local treatment and patients with carcinoma *in situ* (intraepithelial cancer) or lesions equivalent to intramucosal cancer that can be treated endoscopically may be enrolled
• Pregnant or breastfeeding women
• Serious complication
• Positive for hepatitis B surface antigen or positive for hepatitis C virus antibody
• Human immunodeficiency virus (HIV) antibody-positive (a patient may enroll even if HIV antibody has not been tested)
• Patients who do not intend to provide specimens for the ‘Research on Genetic Profiling and Clinical Significance Using Clinical Specimens from Cancer Patients’ (CONDUCTOR Study), in which whole genome analysis will be carried out based on the ‘Whole Genome Analysis Implementation Plan’
• Known microsatellite instability-high or deficient mismatch repair
• The study doctor deemed that the patient is ineligible for this study

AV, anal verge; ECOG PS, Eastern Cooperative Oncology Group performance status; EMVI, extramural venous invasion; MRF, mesorectal fascia.

### Follow-up

Patients undergoing NOM follow-up or TME will undergo imaging, which includes full chest/abdominal/pelvic computed tomography (CT), sigmoidoscopy, and MRI (not essential in those with TME) every 4 months for 2 years from the NOM decision or TME date and every 6 months for 2-5 years from the NOM decision or TME date. Patients with a sustained response compared with the previous evaluation will continue with the NOM follow-up. Patients with progressive disease in relation to their previous evaluations will undergo TME. Biopsies of the primary tumor sites for patients in the NOM follow-up may be carried out as clinically indicated. A new positive biopsy result for adenocarcinoma will be considered a tumor recurrence. QoL will be assessed using the low anterior resection syndrome score, European Organization for the Research and Treatment of Cancer Quality of Life Questionnaire, and 36-Item Short-Form Survey version 2. QoL surveys will be conducted at six different points: (i) trial registration, (ii) restaging after completion of the TNT, and (iii) follow-up periods at 4, 12, 24, and 36 months.

### Outcomes

The primary endpoint will be organ preservation-adapted disease-free survival (DFS),^3^ which is defined as the time from randomization to one of the following events: no resection of the primary tumor due to progression; non-radical surgery of the primary tumor (R2 resection excluding circumferential resection margin positives); locoregional recurrence after R0/1 resection of the primary tumor; non-salvageable local regrowth in case of NOM (no salvage operation or R2 resection); metastatic disease before, at, or after surgery or NOM; second primary colorectal or other cancer; or death (all causes), whichever occurs first. The definitions of each event and event occurrence dates are presented in Table 2.

**Table 2:** Definition of primary endpoint: organ preservation for disease-free survival

Event	Event date
No resection of primary tumor due to local progression or patient unfit for surgery	Date of scheduled, but not performed surgery
Non-radical resection of primary tumor (R2 resection)	Date of surgery
Locoregional recurrence after R0/1 resection of the primary tumor	Date of locoregional recurrence
Non-salvageable local regrowth in case of non-operative management (NOM) (no operation or R2 salvage resection)	Date of diagnosis of non-salvageable regrowth or date of R2 salvage surgery
Any distant metastatic disease before, at, or after surgery or NOM	Date of distant metastases
Second primary colorectal cancer	Date of second colorectal primary
Second primary, other cancers	Date of second primary, other cancers
Death (treatment-related, from same cancer, from other cancer, non-cancer related)	Date of death

The secondary endpoints are presented in Table 3. These endpoints will include surgical morbidity and complications, clinical response, distant metastasis-free survival, local recurrence-free survival, overall survival (OS), TME-free survival, QoL, and functional outcomes based on the treatment arm and surgical procedures. Furthermore, whole-genome sequencing (WGS) and transcriptome sequencing by next-generation sequencing will be carried out to profile LARC before TNT. Moreover, we will evaluate the feasibility of using circulating tumor DNA and whole transcriptome profiles’ extracted exosomes in plasma (liquid biopsy using Caris Assure®), to monitor tumor response to TNT, in patients with LARC treated in both protocol arms. Finally, we will develop a multimodal DL model using the genomic data from WGS/TS and liquid biopsy using Caris Assure®, colonoscopy, pelvic MRI at each point, and clinicopathological features to determine the patients who will benefit from NOM after TNT.

**Table 3:** Secondary endpoints

Secondary endpoints

Clinical complete response (cCR) rate
Clinical response (cCR + near CR) rate
Non-operative management (NOM) selection ratio
Recurrence type and recurrence rate
Distant metastasis-free survival
Local recurrence-free survival (LRFS)
Overall survival
Total mesorectal excision (TME)-free survival
TME-free disease-free survival
Protocol completion rate
Relative dose intensity
Quality of life (QoL) valuation using a QoL questionnaire to assess the following items: low anterior resection syndrome score, European Organization for the Research and Treatment of Cancer Quality of Life Questionnaire, 36-Item Short-Form Survey
Incidence of preoperative treatment-related adverse events determined by the Common Terminology Criteria for Adverse Events version 5.0
**Endpoints in the surgical subgroup**
Pathological complete response rate
Radical resection rate
LRFS
Incidence of postoperative adverse events determined by the Clavien–Dindo classification version 2.0
**Endpoints in subgroups with NOM**
Local regrowth rate
Time to local regrowth
Proportion of salvage surgery in local regrowth cases
Time until salvage surgery
Surgery-related adverse event rate determined by the Clavien–Dindo classification version 2.0 in salvage surgery cases
Proportion of radical resection in salvage surgery cases

### Planned sample size and study period

To detect a decrease in 3-year cumulative probability of organ preservation-adapted DFS from 75.0% to 81.7%, corresponding to a target hazard ratio of 0.70, 608 patients (196 events) would be needed to achieve 70% power at a two-sided α significance level of 0.05. This calculation assumes a 1% drop-out per year, a 4-year enrollment period, and a 3-year follow-up period. Two years after enrollment, sample size recalculation will be planned to improve the statistical power under blinding. The trial start date was 31 October 2022. The enrollment period is from 31 October 2022 to December 2026 (4 years and 2 months), and the follow-up period is 3 years after the end of enrollment.

### Statistical methods

Efficacy will be analyzed using the intention-to-treat population, which includes all randomized populations and excludes patients registered as duplicates or ineligible. The primary hypothesis for organ preservation-adapted DFS will be compared between treatment arms using a stratified log-rank test with stratification factors of randomization except for the institutions (i.e*.* clinical T stage, clinical N status, and distance from the anal verge). Hazard ratios will be estimated using a stratified Cox proportional hazards regression model with the same stratification factors of the log-rank test. Subgroup analysis of clinical T stage, clinical N status, distance from the anal verge, and others [sex, age (<65 versus ≥65 years), ECOG PS, lateral lymph node metastasis, extramural venous invasion, circumference resection margin evaluated using MRI, *RAS* status (wild-type versus mutant), and *BRAF* status (wild-type versus mutant)] will be planned. Other analyses will be described in the statistical analysis plan of this study. Safety will be analyzed in all randomly assigned patients who received at least one dose of the study medication according to the treatment received. Patient-reported outcomes will be analyzed in all patients for whom at least one patient-reported outcome assessment is available and who have received at least one dose of the study treatment.

### Translational research

Primary tumor tissues for translational research, including genomic profiling of WGS/TS, will be collected at three different time points: (i) preoperative biopsy, (ii) TME, and (iii) local regrowth and/or recurrence. Moreover, peripheral blood samples for liquid biopsy using Caris Assure™ will be stored at nine different points: (i) at trial registration, (ii) at restaging after completion of TNT, (iii) 1 month after NOM decision or TME date, and (iv) every 4 months until 2 years from the NOM decision or TME date (4, 8, 12, 16, 20, and 24 months). Moreover, additional blood samples will be collected at local regrowth and/or recurrence. Caris Assure™ is a circulating nucleic acid sequencing, a novel liquid biopsy molecular profiling method that analyzes circulating cell-free DNA and RNA (cfDNA, cfRNA) and genomic DNA and RNA (gDNA, gRNA) from circulating white blood cells.

### Integrated analysis of spatiotemporal trans-omics using artificial intelligence and deep learning

The data collected in this trial (including clinical data; CT, MRI, and colonoscopic images; pathological images; WGS/TS, liquid biopsy, and QoL data) will be integrated into a single database. To identify the predictive markers for the response to TNT, the decision of TME or NOM, and the prediction of recurrence or regrowth, we plan spatiotemporal, trans-omics analyses using artificial intelligence (AI)- and DL-driven genomics, transcriptomics, radiomics, pathomics, colonoscopic images, QoLs, and clinical features.

### Committee associated with the trial

#### Non-operative management committee

The NOM committee was established for consultation when the co-investigator in charge of the facility is unsure of (i) staging before enrollment, (ii) restaging evaluation, and (iii) local regrowth during the NOM. The committee evaluates the data of the applicable case and provides appropriate advice to the co-investigator based on the results of the evaluation.

#### Radiation therapy committee

The committee evaluates whether the SCRT plan at the site meets all dose constraints specified in the protocol. If any value is outside the acceptable limits, replanning is requested and the SCRT plan is again evaluated by the radiotherapy committee members.

#### Pathology central judging committee

To reduce bias and maintain uniformity in pathology diagnosis among centers, a pathology expert will perform a central determination of the pathology diagnosis independent of the co-investigator in charge of the facility.

#### Central judgment committee for pelvic MRI images

For quality control of pelvic MRI image judgment, a diagnostic radiology expert will perform a central judgment of the images, independent of the co-investigator in charge of the facility. Central judgment will not be involved in the judgment at the time of restaging.

#### Colonoscopy image central judgment committee

For the quality control of colonoscopic image judgment, an expert in clinical oncology will perform a central judgment of the images independent of the co-investigator in charge of the facility. Central judgment will not be involved in the judgment at the time of restaging.

## Conclusion

We described the protocol of the ENSEMBLE trial to investigate the efficacy and safety of SCRT followed by CAPOXIRI as a TNT for LARC. This is the first phase III trial to evaluate the effects of preoperative chemotherapy intensity following SCRT on long-term outcomes. We believe that the proposed treatment will improve the survival and preserve the QoL of patients with LARC.

## CRediT author statement

All authors: conceptualization, methodology, and writing-review & editing. Jun Watanabe, Yoshinori Kagawa: writing original draft. Koji Oba: formal analysis. Jun Watanabe, Yoshinori Kagawa, Koji Ando, Daisuke Kotani, Hideki Bando, Mamoru Ueyama, Kay Uehara, Masaaki Ito, Eiji Oki, Ichiro Takemasa, Takeshi Kato: investigation. Takayuki Yoshino: funding acquisition. Jun Watanabe, Yoshinori Kagawa, Takeshi Kato, Takayuki Yoshino: supervision. Jun Watanabe, Yoshinori Kagawa, Koji Ando, Daisuke Kotani, Hideki Bando: project administration.
