# Supplementary material for: Phase III trial of short-course radiotherapy followed by CAPOXIRI versus CAPOX in locally advanced rectal cancer: the ENSEMBLE trial
Source: ESMO Gastrointest Oncol. 2023 Nov 7;1:9–14. doi: 10.1016/j.esmogo.2023.08.002 (PMC12836718; doi:10.1016/j.esmogo.2023.08.002)
Supplement: Supplementary material [file mmc1.docx]

**SUPPLEMENTARY MATERIAL *AND/OR* ADDITIONAL INFORMATION**

***More on background and rationale***

Over the past few decades, the standard treatments for LARC have been preoperative CRT, TME, and variably per institution postoperative adjuvant chemotherapy. These treatment strategies lead to improvement in local disease control in most patients with LARC. However, in nearly one-third of patients, it is difficult to control distant metastasis that is clinically hidden at the time of surgery, resulting in no improvement in OS.^4,5^ In addition, several patients experience functional sequelae after rectal resection, which greatly impair their QoL.^6^ Thus, the two key concerns in treating LARC are as follows: (1) prolonging survival by reducing distant metastases and (2) maintaining QoL in surviving patients by safely avoiding rectal resection.

To resolve these issues, in recent years, TNT, a preoperative combination of CRT or SCRT and systemic chemotherapy, has been developed as a multidisciplinary treatment for LARC. The PRODIGE 23 and RAPIDO randomized phase III trials demonstrated significant improvements in the primary endpoints of DFS and disease-related treatment failure compared with standard CRT and surgery with or without adjuvant chemotherapy.^7,8^ Furthermore, the STELLAR trial from China, which was the third randomized phase III trial showing TNT to be noninferior to standard preoperative CRT in DFS, demonstrated better 3-year OS in the TNT group.^9^ In addition, these pivotal trials showed significantly higher tumor response than standard preoperative CRT. Pathological complete response (pCR) rates of 28% with TNT and 14% with standard CRT in the RAPID trial were similar to those in the PRODIGE 23 (27.8% versus 12.1%) and STELLAR (22.5% versus 12.6%, pCR and sustained clinical CR rate) trials.^7-9^ These results indicate that TNT can yield equal or better locoregional control and distant metastasis than standard preoperative CRT with increasing tumor response and may prolong OS in patients with LARC. Moreover, further insights into how TNT sequences [induction chemotherapy (INCT) followed by CRT and CRT followed by CNCT] affect these outcomes have been obtained from the CAO/ARO/AIO-12 and OPRA randomized clinical trials.^10,11^ In both trials, although there was no difference in long-term outcomes between the two sequences, in the CAO/ARO/AIO-12 trial, pCR rate was significantly higher in the CRT followed by CNCT group than in the INCT followed by CRT group (25% versus 17%) and in the OPRA trial, local regrowth rate was significantly lower in the CRT followed by CNRT group than in the INCT followed by CRT group among patient with non-operative management (NOM) (27% versus 40%). These results suggest that local tumor response depends more on chemoradiation and the interval between radiation and response assessment rather than on the timing of chemotherapy, which may have led to inferior organ preservation rates in patients with NOM in the INCT followed by CRT group.^11,12^ Therefore, considering NOM, CRT followed by CNCT can enhance local tumor regression, lead more patients to complete clinical response (cCR) or near-cCR (nCR), and offer more maintenance of anorectal function and QoL.

The debate regarding the optimal regimen of CRT followed by CNCT has remained in the following two points: (1) radiation dose [long-course radiotherapy (LCRT) [45-50.4 Gy/25-28 fractions (fx)] versus SCRT (25 Gy/5 fx)] and (2) chemotherapy intensity (doublet versus triplet). In terms of radiation dose, the ACO/ARO/AIO-18.1 randomized phase III trial is ongoing (NCT04246684).^13^ In this phase III trial, SCRT followed by FOLFOX/ CAPOX according to the RAPIDO trial and LCRT followed by FOLFOX/CAPOX according to CAO/ARO/AIO-12 were directly compared. Moreover, in both arms, according to restaging after completion of TNT for patients achieving a cCR, the NOM option with close follow-up is scheduled. In contrast, the use of triplet regimens of TNT has been investigated for their higher response rates and improved prognosis.^14^ The PRODIGE 23 trial randomized patients with LARC to 3 months of neoadjuvant modified folinic acid, fluorouracil, irinotecan hydrochloride, and oxaliplatin (mFOLFIRINOX), LCRT, and TME and 3 months of postoperative adjuvant chemotherapy or standard treatment (LCRT + TME + 6 months of postoperative adjuvant FOLFOX).^7^ The experimental group had a significantly higher pCR rate (28% versus 12%, *P* < 0.001), indicating the efficacy of the TNT regimen, including the triplet regimen. In addition, the JANUS phase II rectal cancer trial, a randomized trial in which patients with LARC undergoing LCRT are randomized to a combination of modified FOLFOX, CAPOX, or mFOLFIRINOX with cCR as the primary endpoint, is currently ongoing (NCT05610163). However, no phase III trials have compared triplet and doublet regimens followed by SCRT for TNT. As a triplet regimen, CAPOXIRI has been developed for the convenience of not requiring a central venous port and to reduce neutropenia. In the Japanese QUATTRO-II trial, capecitabine (1600 mg/m^2^), oxaliplatin (130 mg/m^2^), irinotecan (200 mg/m^2^), and bevacizumab (7.5 mg/kg) have been considered safe.^1^ Therefore, CAPOXIRI, which has already been well-tolerated in Japanese patients, is expected to be a promising TNT regimen for LARC. Therefore, we designed the ENSEMBLE trial to compare CNCT intensity after SCRT with that after CAPOXIRI as a triplet regimen.

In addition, despite the higher response of TNT, approximately one-fifth of patients have incomplete CR (iCR) and poor prognosis.^9^ As a longer interval before surgery after completing preoperative CRT is associated with worse OS and DFS in tumors with a poor response,^15^ it is also necessary to predict the responders and non-responders to TNT. However, no predictive biomarkers have yet been established for LARC. Therefore, to explore predictive biomarkers to estimate the response to TNT and identify patients who would benefit from NOM after TNT, we planned TR using multi-omics data, including WGS/TS, liquid biopsy, radiomics, pathomics, and clinical features by DL with AI.

**REFERENCES**

4. Hofheinz R-D, Wenz F, Post S, et al. Chemoradiotherapy with capecitabine versus fluorouracil for locally advanced rectal cancer: a randomised, multicentre, non-inferiority, phase 3 trial. *Lancet Oncol*. 2012;13(6):579-588.

5. Schmoll H-J, Stein A, Cutsem EV, et al. Pre- and postoperative capecitabine without or with oxaliplatin in locally advanced rectal cancer: PETACC 6 trial by EORTC GITCG and ROG, AIO, AGITG, BGDO, and FFCD. *J Clin Oncol*. 2021;39(1):17-29.

6. Gavaruzzi T, Lotto L, Giandomenico F, Perin A, Pucciarelli S. Patient-reported outcomes after neoadjuvant therapy for rectal cancer: a systematic review. *Expert Rev Anticancer Ther.* 2014;14(8):901-918.

7. Conroy T, Bosset J-F, Etienne P-L, et al. Neoadjuvant chemotherapy with FOLFIRINOX and preoperative chemoradiotherapy for patients with locally advanced rectal cancer (UNICANCER-PRODIGE 23): a multicentre, randomised, open-label, phase 3 trial. *Lancet Oncol*. 2021;22(5):702-715.

8. Bahadoer RR, Dijkstra EA, van Etten B, et al. Short-course radiotherapy followed by chemotherapy before total mesorectal excision (TME) versus preoperative chemoradiotherapy, TME, and optional adjuvant chemotherapy in locally advanced rectal cancer (RAPIDO): a randomised, open-label, phase 3 trial. *Lancet Oncol*. 2021;22(1):29-42.

9. Jin J, Tang Y, Hu C, et al. Multicenter, randomized, phase III trial of short-term radiotherapy plus chemotherapy versus long-term chemoradiotherapy in locally advanced rectal cancer (STELLAR). *J Clin Oncol*. 2022;40(15):1681-1692.

10. Fokas E, Schlenska-Lange A, Polat B, et al. Chemoradiotherapy plus induction or consolidation chemotherapy as total neoadjuvant therapy for patients with locally advanced rectal cancer. *JAMA Oncol*. 2022;8(1):e215445.

11. Garcia-Aguilar J, Patil S, Gollub MJ, et al. Organ preservation in patients with rectal adenocarcinoma treated with total neoadjuvant therapy. *J Clin Oncol*. 2022;40(23):2546-2556.

12. Kazi M, Saklani A. Total neoadjuvant therapy for all rectal cancers: is this the way ahead of the OPRA trial? *J Clin Oncol*. 2022;41(2):415-416.

13. Slevin F, Hanna CR, Appelt A, et al. The long and the short of it: the role of short-course radiotherapy in the neoadjuvant management of rectal cancer. *Clin Oncol (R Coll Radiol)*. 2022;34(5):e210-e217.

14. Pollom EL, Shelton A, Fisher GA, et al. Phase II trial of organ preservation program using short-course radiation and folfoxiri for rectal cancer (SHORT-FOX). *J Clin Oncol*. 2022;40(suppl 4):TPS218-TPS218.

15. Deidda S, Elmore U, Rosati R, et al. Association of delayed surgery with oncologic long-term outcomes in patients with locally advanced rectal cancer not responding to preoperative chemoradiation. *JAMA Surg*. 2021;156(12):1141-1149.
